# A prospective single-arm study on the relationship between dose-volume parameters of pelvic functional bone marrow and acute hematological toxicities during intensity-modulated radiotherapy with or without concurrent chemotherapy for uterine cervical/endometrial cancer

**DOI:** 10.1186/s13014-023-02380-8

**Published:** 2023-11-27

**Authors:** Hongbo Chen, Qian Zhong, Yujie Liu, Jinyan Li, Wenjing Deng, Jie Wang, Shuquan Zhou, Zengrong Yu, Xianzhan Huang, Yuanqiong Huang, Bo Zhen, Jihong Wei, Weijian Zhang, Xiaohong Ruan, Lin Xiao

**Affiliations:** 1 Department of Radiotherapy Center, Jiangmen Central Hospital, Jiangmen, Guangdong Province China; 2 Department of Oncology Unit II, Jiangmen Central Hospital, Jiangmen, Guangdong Province China; 3 Department of Gynecology, Jiangmen Central Hospital, Jiangmen, Guangdong Province China; 4 Department of Oncology Unit III, Jiangmen Central Hospital, Jiangmen, Guangdong Province China; 5 Department of Oncology, The First People’s Hospital of Liangshan Yi Autonomous Prefecture, Xichang, Sichuan Province, China

**Keywords:** Uterine cervical cancer, Endometrial cancer, Hematologic toxicity, Chemoradiotherapy, Functional bone marrow

## Abstract

**Background:**

FLT-PET/CT can accurately identify and locate functional bone marrow (FBM) with hematopoietic capability, the FBM were divided into two levels as FBM_1_ (strongest hemopoietic ability region)and FBM_2_ (moderate hemopoietic ability region) via FLT-PET/CT. The purpose of this study was to explore the relationship between dose-volume parameters of pelvic FBM and hematologic toxicity (HT) during radiotherapy with or without concurrent chemotherapy for uterine cervical/endometrial cancer.

**Methods:**

From December 2016 to September 2021, ninety-seven uterine cervical/endometrial cancer patients received intensity-modulated radiation therapy were prospectively recruited in this single-arm, prospective, phase II trial. Blood counts were reviewed weekly during radiotherapy. Single- and multifactor regression methods were used to analyze the relationships between dose-volume parameters of FBM_1/2_ and grade ≥ 2 HT. ROC curves were used to determine the cutoff values for the dose-volume parameters of FBM_1/2_.

**Results:**

The incidence of grade ≥ 2 leukopenia, neutropenia, thrombocytopenia and anemia in patients during radiotherapy was 63.9%, 45.4%, 19.6% and 38.8% respectively, and the median occurrence time was the 29th, 42th, 35th and 31th day, respectively. Multivariate regression analysis showed that the D_max_ of FBM_1_ was significantly related to grade ≥ 2 leukopenia (OR = 1.277 95% CI 1.067–1.528, *P* = 0.008), D_mean_ of FBM_2_ was significantly related to grade ≥ 2 thrombocytopenia (OR = 1.262 95% CI 1.066–1.494, *P* = 0.007), and V_10_ of FBM_1_ was significantly related to grade ≥ 2 anemia (OR = 1.198 95% CI 1.003–1.431, *P* = 0.046). The incidence of grade ≥ 2 leukopenia for patients with FBM_1_ D_max_ < 53 Gy was lower than that for patients with FBM_1_ D_max_ ≥ 53 Gy (53.4% vs. 95.8%, *P* < 0.001). The incidence of grade ≥ 2 thrombocytopenia in patients with FBM_2_ D_mean_ < 33 Gy was lower than that in patients with FBM_2_ D_mean_ ≥ 33 Gy (0 vs. 28.4%, *P* < 0.001). The incidence of grade ≥ 2 anemia for patients with FBM_1_ V_10_ < 95% was lower than that in patients with FBM_1_ V_10_ ≥ 95% (24.4% vs. 57.1%, *P* = 0.003).

**Conclusions:**

Grade ≥ 2 HT usually occurs in the 4th week of radiotherapy for patients with uterine cervical/endometrial cancer. The D_max_ and V_10_ of FBM_1_ and the D_mean_ of FBM_2_ were significantly associated with the occurrence of grade ≥ 2 HT. The recommended optimal dose constraints were FBM_1_ D_max_ < 53 Gy, V_10_ < 95%, and FBM_2_ D_mean_ <33 Gy.

**Supplementary Information:**

The online version contains supplementary material available at 10.1186/s13014-023-02380-8.

## Background

Radiotherapy is one of the major treatments for uterine cervical cancer and endometrial cancer. Hematological toxicity (HT) is a common adverse event during pelvic radiation, and concurrent chemoradiotherapy further increases the risk of HT [1]. Serious HT causes a series of problems. First, HT lowers patient tolerance to radiotherapy and chemotherapy [2], extends treatment duration and affects oncological outcome. Second, the use of hematopoietic growth factors such as granulocyte colony-stimulating factor (G-CSF), thrombopoietin (TPO), interleukin-11 (IL-11), erythropoietin (EPO) to treat myelosuppression not only increases the patients’ economic burden but also may accelerate the aging of hematopoietic stem cells and induce long-term bone marrow damage caused by radiation [3]. Third, HT increases the risk of subsequent infection and death.

Some low dose-volume parameters (such as V_10_ and V_20_) [4–8] and high dose-volume parameters (such as V_40_) of the pelvic bone marrow have been related to acute HT [7–10]. Intensity-modulated radiation therapy (IMRT) can reduce the irradiated volume of the pelvic bone marrow [6, 11, 12] and reduce the risk of HT [4, 13, 14]. However, there is no consensus on delineating the pelvic bone marrow or reliable and recognized dose-volume parameters of the pelvic bone marrow to guide clinical practice. Most studies have classified the whole pelvic bone as an organ at risk (OAR), but the dose-volume parameters of the pelvic bone marrow to be recommended were significantly different.

Accurate identification of functional bone marrows (FBMs) with hematopoietic function and taking measures to prevent them from extra irradiating is the most effective way to reduce the risk of HT during radiotherapy or chemoradiotherapy. A series of studies on 18 F-fluorothymidine positron emission tomography (FLT-PET) have shown that the hematopoiesis capacities of pelvic bone marrow are heterogeneous at different pelvic anatomical sites, and this information can help optimize IMRT treatment planning [15–17]. McGuire et al. [17] used FLT-PET to accurately locate the distribution of pelvic FBMs. According to the difference in the FLT standard uptake value (SUV), the hematopoietic functional pelvic bones were divided into two levels. The regions with the strongest activity of hematopoiesis (SUV 3.3 ~ 3.7) included the bilateral iliac ala above the sacroiliac joint and inside of the straight line passing through the middle of the acetabulum and the narrowing of the iliac bone, the fifth lumbar vertebra and the sacrum above the sacroiliac joint, which was classified as the first level and named FBM_1_. Additionally, the other regions with hematopoiesis activity (SUV 2.6 ~ 2.9) included the bilateral ilium from the lower edge of the sacroiliac joint to the upper edge of the femoral head, the area of the pubis and the ischium below the upper edge of the bilateral femoral heads and inside of the straight line passing through the outer boundary of obturator foramen, which was classified as the secondary level and named FBM_2_.

To date, there are no any reports on the relationship between HT and the dose-volume parameters of FBM_1_ and FBM_2_ during radiotherapy or chemoradiotherapy. The purpose of this study was to prospectively explore the relationship between the dose-volume parameters of pelvic FBM_1/2_ and the risk of grade ≥ 2 HT during IMRT with or without concurrent cisplatin for uterine cervical and endometrial cancer. The preliminary results [18] of this study were presented as a poster during the 2020 ASTRO annual meeting (#3066), and updated results are reported in this article.

## Methods

### Study design

This study is a single-center, single-arm, prospective, phase II clinical trial (registration ID number: ChiCTR-OIN-17,011,113, funded by Guangdong Province Medical Science and Technology Research Foundation, No. A2016623). This study prospectively recruited uterine cervical cancer and endometrial cancer patients who needed radiotherapy with or without concurrent chemotherapy from December 2016 to September 2021 in Jiangmen Central Hospital, Guangdong Province, China. Clinical staging of uterine cervical cancer or endometrial cancer was based on the FIGO 2018 guidelines. Before treatment, all patients underwent pelvic MRI, blood counts, liver and kidney function tests, thoracic and abdominal CT, and serum tumor marker (such as CEA, CA125, SCC, etc.) assessments.

The inclusion criteria were as follows: (1) uterine cervical cancer/endometrial cancer confirmed by pathological diagnosis treated by radical chemoradiotherapy or adjuvant radiotherapy with or without concurrent chemotherapy; (2) age 18–80, PS score 0–2, and normal hepatic and renal function tests; (3) Adequate bone marrow function before radiotherapy (regardless of whether they receive induction chemotherapy or not) indicated by, WBC ≥ 3 × 10^9/L, neutrophils ≥ 1.5 × 10^9/L, hemoglobin ≥ 8.0 g/dL, and platelet ≥ 75 × 10^9/L, as for patients received chemoradiotherapy, the requirements indicated by, WBC ≥ 3 × 10^9/L, neutrophils ≥ 1.5 × 10^9/L, hemoglobin ≥ 9.0 g/dL, and platelet ≥ 100 × 10^9/L; and (4) provided signed informed consent forms to receive radiotherapy or chemoradiotherapy.

The exclusion criteria were as follows: (1) para-aortic field irradiation; (2) previous pelvic radiotherapy; (3) serious underlying disease or inability to tolerate radiotherapy or chemoradiotherapy; (4) fewer than four times of blood counts during radiotherapy and no any test of blood counts within one month after radiotherapy and (5) not finishing the whole radiation plan.

The sample size of this study was estimated by referring to the target value method for single-group, nonrandomized controlled clinical trials. According to the preliminary results and literature reports, the probability of grade 2 HT of uterine cervical/endometrial cancer during concurrent chemoradiotherapy is approximately π_0_ = 23%. In the intervention group, the probability of grade 2 HT may be π_1_ = 13%. For a one-sided α = 0.05, β = 0.25, δ = π_0_-π_1_ = 0.10, µα = 1.65 and µβ = 0.68; when the parameters are substituted into the following formula:

$$\text{N}={\pi }0\times \left(1-{\pi }0\right)\times {\left[\frac{\left(\mu \alpha +\mu \beta \right)}{\delta }\right]}^{2}$$

n = 0.23 × 0.77 × 23.3 × 23.3 = 96 cases.

### Radiation treatment

The patients drank 250 ml iodine contrast medium diluent (1:50) half to one hour before CT scanning, and were immobilized with a vacuum bag in the supine position or with an Orfit frame in the prone position. The CT scan ranged from the lower edge of the second lumbar vertebra to 5 cm below the ischial tuberosity, with a slice thickness of 5 mm. All patients underwent plain and contrast enhanced scanning, except for those who were allergic to the iodine medium. The two sets of CT images were transferred to the Eclipse 10.0/15.5 treatment planning system (TPS) workstation for target delineation. The gross target volume (GTV) was defined as the primary cervical/uterine carcinoma, and GTVnd was defined as the pelvic metastatic lymph nodes. For postoperative adjuvant radiotherapy, delineation of the clinical target volume (CTV) for primary cervical/uterine carcinoma and local or regional pelvic lymph node was performed according to the RTOG recommendations from 2008 [19]. The vaginal cuff or vaginal stump was routinely included as primary cervical/uterine carcinoma CTV for adjuvant radiotherapy. For radical radiotherapy, delineation of the CTV for primary cervical/uterine carcinoma was performed according to the RTOG recommendations from 2011 [20]. The planning target volume (PTV) was created with a 7 ~ 9 mm margin around the CTV, and PGTVnd was created with a 7 ~ 9 mm margin around the GTVnd. Generally, we did not give any concomitant boost doses to the primary tumors of the cervix or endometrium during external irradiation for radical radiotherapy, except in patients with positive pelvic lymph nodes and parametrial infiltration. The prescribed dose for the PTV of the IMRT plan was 45-50.4 Gy/1.8 Gy× (25-28 F). If patients had positive lymph nodes or positive parametrial margin, a concomitant boost dose was given to these areas, and the prescribed dose for PGTV/PGTVnd was 60–66 Gy/28 F. IMRT plans for all patients covered 5–7 fields, with doses administered once a day and 5 days a week. The 98% prescription isodose line should cover more than 98% of the PTV for all IMRT treatment plans, and the maximum dose point should not exceed 110% of the prescribed dose. The PTV volume receiving ≤ 93% of the prescribed dose should be less than 1%. The radiation energy was 6 MV. All IMRT plans were designed in Eclipse 10.0/15.5, and radiotherapy treatment was performed on a Varian clinac ix or EDGE linear accelerator. For radical radiotherapy, patients received brachytherapy of 6 Gy per fraction ×5 F (or 7 Gy per fraction ×4 F) after finishing external irradiation.

**Fig. 1:**
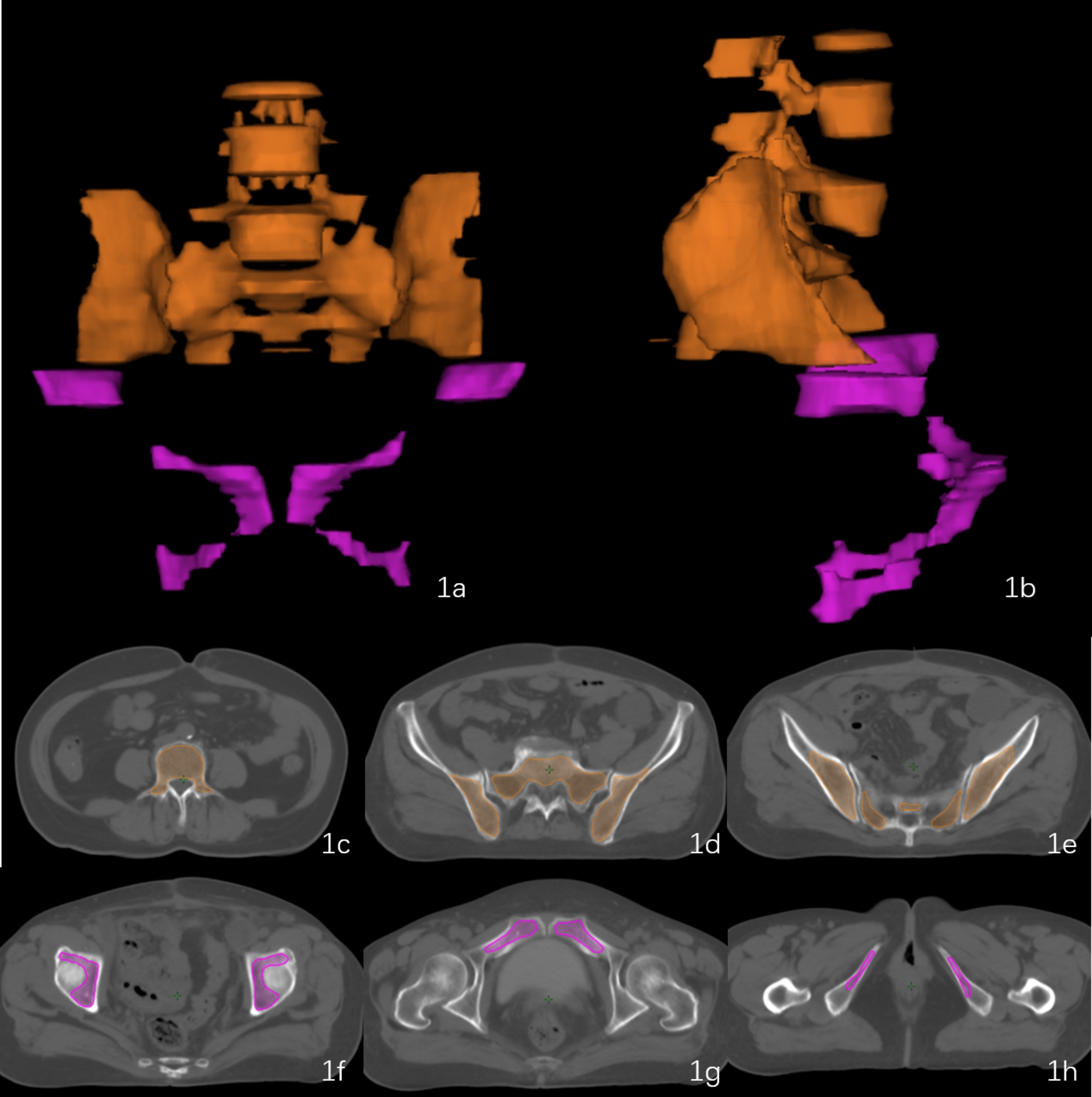
Delineation range of FBM_1/2_. (**1**) Three-dimensional imaging of FBM_1/2_ (1a, 1b). (**2**) The orange areas represent the delineation range of FBM_1_ (1c, 1d, 1e). (**3**)The pink areas represent the delineation range of FBM_2_ (1f, 1 g, 1 h)

Pelvic FBM_1/2_ was defined in accordance with the methods proposed by McGuire SM et al. [17] and was delineated with a CT window width of 2000 Hu and a window level of 800 Hu (Fig. 1). Additionally, the delineation and dose constraints of other organs at risk(OARs), such as the rectum, small intestine, bladder and bilateral femoral heads, were performed according to the RTOG 0418 recommendations [9] and combined it with the experience of our radiotherapy center. Our dose constraints for OARs are as follows, small intestine V40 ≤ 30%, V35 ≤ 180 cm³, V40 ≤ 100 cm³, bowel bag V40 ≤ 30%, rectum V45 ≤ 65%, bladder V45 ≤ 50%, femoral head Dmax ≤ 50 Gy, V30 ≤ 15%, spinal cord Dmax ≤ 42 Gy.The recommended dose constraints for FBM_1_ and FBM_2_ were as follows: V_5_ ≤ 90%, V_10_ < 80%, and D_mean_<2800–3000 cGy. The target coverage and conformability were prioritized, meanwhile balance between target coverage and the dose constraint requirements of important OARs such as the rectum, bladder, colon, small intestine, and femoral head, was achieved for every final treatment plan. Finally, the dose constraints of FBM_1/2_ were pursued as far as possible, and not mandatory to meet all requirements.

### Chemotherapy regimens

Neoadjuvant chemotherapy (1–4 courses) was used as appropriate for uterine cervical/ endometrial cancer patients with large tumor size(diameter ＞ 5 cm) and central necrotic lesions receiving radical radiotherapy. In addition, for patients with high-risk postoperative pathological factors or stage III-IV, neoadjuvant chemotherapy before radiotherapy may be used as appropriate. The main chemotherapy regimen was paclitaxel and cisplatin or carboplatin, if the pathological type was neuroendocrine carcinoma, the chemotherapy regimen was etoposide and cisplatin or carboplatin.

The indications for concurrent chemotherapy for uterine cervical cancer were as follows (1) treatment with radical radiotherapy (2) treatment with adjuvant radiotherapy with postoperative high-risk factors, pelvic lymph node metastasis, positive surgical margin, and parametrial invasion.

The indications for concurrent chemotherapy for endometrial cancer were as follows 1. treatment with radical chemoradiotherapy2. treatment with adjuvant radiotherapy with pelvic lymph node metastasis, invasion into the uterine serosal layer, ovary/fallopian tube, vagina and parametrial tissue, bladder or rectum.

Concurrent chemotherapy regimen for uterine cervical cancer/endometrial cancer patients was single-drug cisplatin (40 mg/m²) once a week until the end of radiotherapy.

If grade 2 HT occurred during chemoradiotherapyor radiotherapy, close monitor was taken or G-CSF, IL-11, or EPO was used to treat myelosuppression if necessary, and the next course chemotherapy dose was not reduced. When grade 3 HT occurred, G-CSF, IL-11 or EPO was used to provide supportive treatment, but radiotherapy was not discontinued. After the patients recovered to grade 1–2 HT, the dose of the next course of concurrent chemotherapy was reduced by 25%. When grade 4 HT occurred, radiotherapy or chemoradiotherapy was temporarily interrupted, and active supportive treatment was given to correct bone marrow suppressions. After patients recovered to grade 1–2 HT, radiotherapy was resumed, and subsequent concurrent chemotherapy was terminated.

### Toxicity evaluation and follow-up

Before radiotherapy, blood counts and weight measurements were routinely monitored. During the course of radiotherapy or chemoradiotherapy, blood counts were reviewed weekly, gastrointestinal toxicity and appetite were monitored weekly, and liver and kidney function was reviewed every 1–2 weeks. Within 1 month after the end of radiotherapy, blood counts were reviewed weekly. A medical examination, pelvic MRI examination and relevant blood tests were performed every 3 months after radiotherapy for 2 years and then every 6 months thereafter until 5 years. All hematological events were evaluated according to the Common Terminology Criteria for Adverse Events (CTCAE) v 4.0 criteria, and the most severe graded event was recorded.

### Statistical analysis

Statistical analysis was performed using SPSS 22.0 (SPSS Inc., Chicago, IL, USA). Measurement data are shown as the median, interquartile range (IQR) or range. Count data and ranked data are shown as cases (n) and percentages (%). Univariate regression analysis and the chi-square test were used to analyze the relationships among clinical factors, FBM_1/2_ dose-volume parameters and grade ≥ 2HT. Multivariate regression models (maximum likelihood, forward stepwise regression method) were used to detect clinical factors or dose-volume parameters from univariate analysis in which the *P* < 0.2 was set as a screening threshold value. Receiver operating characteristic (ROC) curves were used to analyze the relationship between FBM_1/2_ dose-volume parameters and HT, the largest Youden index (defined as sensitivity + specificity − 1) was used to determine the cutoff values for dose-volume parameters with statistical significance in multivariate analysis, and the chi-square test was used to verify the difference between groups at the cutoff point. *P* < 0.05 was considered significant.

## Results

### Patient characteristics

**Fig. 2:**
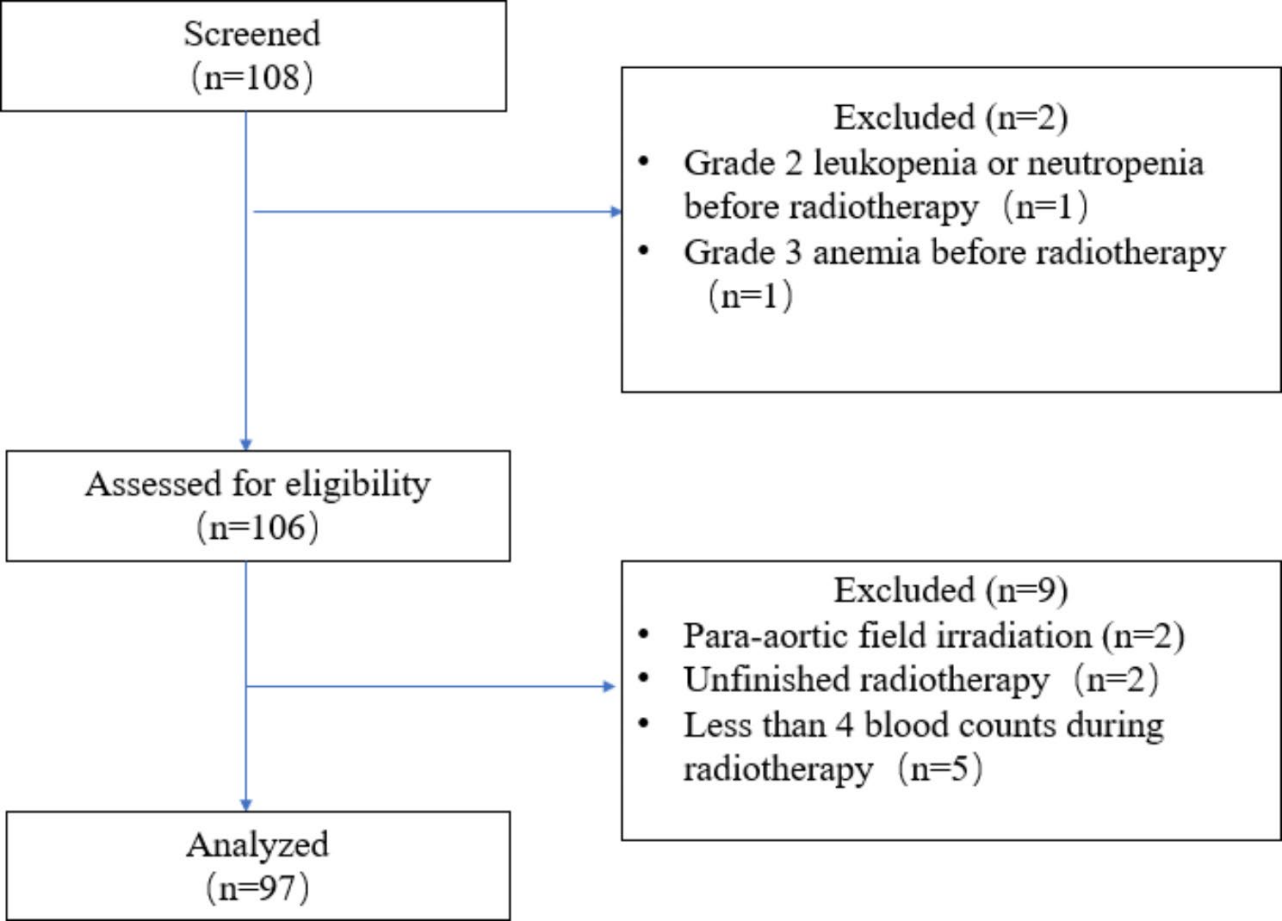
CONSORT flow diagram

From February 2016 to September 2021, 108 uterine cervical cancer/endometrial cancer patients receiving radiotherapy with or without concurrent chemotherapy were recruited at Jiangmen Central Hospital. Finally, 97 patients met the inclusion criteria (Fig. 2). The clinical and tumor characteristics of all patients enrolled in the study are shown in Table 1.

**Table 1:** Clinical and tumor characteristics

Characteristic	Number(%)
Age, year	Median, 52 (Range,33 ~ 79)
Height, cm	Median, 56.6 (Range,140 ~ 168)
Weight, kg	Median, 56 (Range,36 ~ 90)
BMI, kg/m²	Median, 22.43 (Range, 15.6 ~ 34.7)
Types of gynecologic tumors	
Uterine cervical cancer	72(74.2)
Endometrial cancer	25(25.8)
Pathological type of uterine cervical cancer	
Squamous cell carcinoma	58(59.8)
Adenocarcinoma	10(10.3)
Neuroendocrine carcinoma	3(3.1)
Adenosquamous carcinoma	1(1.0)
Stage of uterine cervical cancer	
IA2	1(1.0)
IB1	12(12.4)
IB2	8(8.2)
IB3	1(1.0)
IIA1	2(2.1)
IIA2	9(9.3)
IIB	18(18.6)
IIIA	1(1.0)
IIIA	1(1.0)
IIIC1	19(19.6)
IVA	1(1.0)
Pathological type of endometrial cancer	
Endometrioid adenocarcinoma	20(20.6)
Squamous cell carcinoma	1(1.0)
Clear cell carcinoma	3(3.1)
Papillary serous carcinoma	1(1.0)
Stage of endometrial cancer IA	2(2.1)
IB	4(4.1)
II	9(9.3)
IIIA	4(4.1)
IIIC1	5(5.2)
IVA	1(1.0)
Pathological grade	
G1	3(3.1)
G2	32(33)
G3	38(39.2)
Gx	24(24.7)
Leukopenia before radiotherapy	
0°	83(85.6)
1°	14(14.4)
Neutropenia before radiotherapy	
0°	92(94.8)
1°	5(5.2)
Anemia before radiotherapy	
0°	64(66)
1°	16(16.5)
2°	17(17.5)
Thrombocytopenia before radiotherapy	
0°	96(99)
1°	1(1)

### Treatment

Seventy-nine patients (81.4%) received induction chemotherapy before radiotherapy, with a median of 2 courses (range: 1–4 courses). Sixty-seven patients (69.1%) received concurrent chemotherapy during radiotherapy, with a median of 4 courses (range: 1–7 courses). All patients received IMRT (2 cases with 5 fields and 95 cases with 7 fields). 73 patients (75.3%) received postoperative adjuvant radiotherapy (48 patients for uterine cervical cancer and 25 patients for endometrial cancer, 34 patients with a prescribed dose of 45 Gy/25 F and 39 patients with a prescribed dose of 50.4 Gy/28 F, among which 1 patient had a simultaneous integrated boost dose of 10 to 60 Gy because of positive parametrial invasion, and 3 patients had an additional boost dose of brachytherapy of 6 Gy ×3 F because of residual cancer in the vaginal stump), and the other 24 uterine cervical cancer patients (24.7%) were treated with radical radiotherapy (23 patients with an external pelvic irradiation prescribed dose of 50.4 Gy/28 F and 1 patient with a prescribed dose of 47.6 Gy/28 F, 17 patients of them had a simultaneous integrated boost dose of 10 Gy to total dose 60 Gy to their positive lymph nodes. All patients received brachytherapy of 6 Gy ×5 F(or 7 Gy ×4 F) after finishing external irradiation). The median completion time for radiotherapy was 38 days (IQR: 36.5 ~ 42.5 days). Details of the treatment protocol are shown in S1 in supplementary materials and the dose-volume parameters of FBM_1/2_ are shown in S2 in supplementary materials.

### Occurrence time and distribution of grade ≥ 2 HT and grade ≥ 3 HT

The time distribution of grade ≥ 2 HT and grade ≥ 3 HT that occurred during the course of radiotherapy and after radiotherapy within the first month is shown in Table 2. Grade ≥ 2 leukopenia occurred in 62 patients (63.9%) and the median occurrence time was the 29th day (IQR 21.75-39.5th day, range 5-64th day). Grade ≥ 2 neutropenia occurred in 44 patients(45.4%) and the median occurrence time was the 42th day (IQR 28-49th day, range 11-75th day). Grade ≥ 2 thrombocytopenia occurred in 19 patients (19.6%) and the median occurrence time was the 35th day (IQR 30-42th day, range 20-47th day). Seventeen patients with grade ≥ 2 anemia before radiotherapy were excluded, grade ≥ 2 anemia occurred in 31 patients (38.8%) and the median occurrence time was the 31th day (IQR 21-36th day, range 9-63th day). Grade ≥ 3 leukopenia occurred in 26 patients (26.8%) and the median occurrence time was the 38th day (IQR 31.5-46.25th day, range 20-56th day). Grade ≥ 3 neutropenia occurred in 14 patients (14.4%) and the median occurrence time was the 40th day (IQR 35.75-44.5th day, range 11-64th day). Grade ≥ 3 thrombocytopenia occurred in 11 patients (11.3%) and the median occurrence time was the 35th day (IQR 34-44th day, range 28-47th day). And grade ≥ 3 anemia occurred in 16 patients (16.5%) and the median occurrence time was the 41.5th day (IQR 35-45th day, range 32-55th day).

**Table 2:** Incidence and occurrence time of ≥ grade 2 HT and ≥ grade 3 HT

HT categories	Number(%)	Median(IQR, Range, day)	
Grade ≥ 2 leukopenia	62 (63.9%)	29th day (IQR 21.75-39.5th day, Range 5-64th)	
Grade ≥ 2 neutropenia	44 (45.4%)	42th day (IQR 28-49th day, Range 11-75th)	
Grade ≥ 2 thrombocytopenia	19 (19.6%)	35th day (IQR 30-42th day, Range 20-47th)	
Grade ≥ 2 anemia	31 (38.8%)	31th day (IQR 21-36th day, Range 9-63th)	
Grade ≥ 3 leukopenia	26 (26.8%)	38th day (IQR 31.5-46.25th day, Range 20-56th)	
Grade ≥ 3 neutropenia	14 (14.4%)	40th day (IQR 35.75-44.5th day, Range 11-64th)	
Grade ≥ 3 thrombocytopenia	11 (11.3%)	35th day (IQR 34-44th day, Range 28-47th)	
Grade ≥ 2 anemia	16 (16.5%)	41.5th day (IQR 35-45th day, Range 32-55th)	

### Dose-volume parameters of FBM_1/2_ and clinical factors associated with grade ≥ 2 HT

The dose volume parameters of FBM_1/2_ and clinical factors related to grade ≥ 2 HT were analyzed for leukopenia, neutropenia, thrombocytopenia and anemia. Single-factor analyses are shown in the S3, S4, S5 and S6 in supplementary materials, and multifactor analyses are shown in Table 3.

**Table 3:** Multivariate logistic regression analysis of grade ≥ 2 HT

HT categories	Factors	Odds ratio	95% CI	*P*
Leukopenia	Courses of concurrent chemotherapy	1.513	1.121–2.041	0.007
	D_max_ of FBM_1_	1.277	1.067–1.528	0.008
Neutropenia	Prescribed dose of external pelvic irradiation	1.566	1.161–2.111	0.003
	Courses of concurrent chemotherapy	1.404	1.030–1.912	0.032
	Boost dose to the lymph nodes/ parametrial tissue(No vs. Yes)	6.915	1.084–44.110	0.041
Thrombocytopenia	D_mean_ of FBM2	1.262	1.066–1.494	0.007
	Boost dose to the lymph nodes/ parametrial tissue(No vs. Yes)	12.663	3.515–45.619	＜0.001
Anemia	Age	1.074	1.010–1.143	0.023
	Weight	0.923	0.860–0.991	0.028
	Courses of concurrent chemotherapy	1.784	1.302–2.444	＜0.001
	V_10_ of FBM_1_	1.198	1.003–1.431	0.046

The single factor analysis suggested that the courses of induction chemotherapy before radiotherapy, the courses of concurrent chemotherapy, the prescribed dose of external pelvic irradiation, a boost dose to the lymph nodes/parametrial tissue, V_30_, V_35_, V_40_, V_45_, V_50_, D_max_ of FBM_1_, V_5_, V_10_, V_30_, V_35_, V_40_, V_45_, V_50_, D_max_, and D_mean_ of FBM_2_ were related to grade ≥ 2 leukopenia (*P* < 0.05). The results of the multivariate regression analysis suggested that the courses of concurrent chemotherapy (OR = 1.513, 95% CI 1.121–2.041, *P* = 0.007) and D_max_ of FBM_1_ (OR = 1.277, 95% CI 1.067–1.528, *P* = 0.008) were significantly associated with grade ≥ 2 leukopenia.

The single factor analysis suggested that the courses of induction chemotherapy, the courses of concurrent chemotherapy, the prescribed dose of external pelvic irradiation, a boost dose to the lymph nodes/parametrial tissue, V_40_, V_45_, V_50_, D_max_ of FBM_1_, V_5_, V_10_, V_30_, V_35_, V_40_, V_45_, V_50_, D_max_, and D_mean_ of FBM_2_ were related to grade ≥ 2 neutropenia (*P* < 0.05). Multivariate regression analysis suggested that the prescribed dose of external pelvic irradiation (OR = 1.566, 95% CI 1.161–2.111, *P* = 0.003), the courses of concurrent chemotherapy (OR = 1.404, 95% CI 1.030–1.912, *P* = 0.032) and the boost dose to the lymph nodes/parametrial tissue (OR = 6.915, 95% CI 1.084–44.110, *P* = 0.041) were significantly associated with grade ≥ 2 neutropenia. Patients with a boost dose of lymph nodes/parametrial tissue had a higher risk of grade ≥ 2 neutropenia than those without a boost dose (88.9% vs.35.4%, *P* < 0.001).

The single factor analysis suggested that BMI, the courses of concurrent chemotherapy, the prescribed dose of external pelvic irradiation, a boost dose to the lymph nodes/parametrial tissue, V_40_, V_45_, V_50_, D_max_ of FBM_1_, V_5_, V_25_, V_30_, V_35_, V_50_, D_max_, and D_mean_ of FBM_2_ were related to grade ≥ 2 thrombocytopenia (*P* < 0.05). Multivariate regression analysis suggested that the D_mean_ of FBM_2_ (OR = 1.262, 95% CI 1.066–1.494, *P* = 0.007) and a boost dose to the lymph nodes/ parametrial tissue (OR = 12.663, 95% CI 3.515–45.619, *P* < 0.001) were significantly associated with grade ≥ 2 thrombocytopenia. Patients with a boost dose to the lymph nodes/parametrial tissue had a higher risk of grade ≥ 2 thrombocytopenia than those without a boost dose (55.6% vs.11.4%, *P* < 0.001).

Seventeen patients with grade ≥ 2 anemia before radiotherapy were excluded, and 80 patients were analyzed. The single factor analysis suggested that the courses of induction chemotherapy, the courses of concurrent chemotherapy, the prescribed dose of external pelvic irradiation, a boost dose to the lymph nodes/parametrial tissue, V_10_, V_40_, V_45_, D_max_ of FBM_1_, V_5_, V_10_, V_50_ and D_max_ of FBM_2_ were related to grade ≥ 2 anemia (*P* < 0.05). Multivariate regression analysis suggested that age (OR = 1.074, 95% CI 1.010–1.143, *P* = 0.023), weight (OR = 0.923, 95% CI 0.860–0.991, *P* = 0.028), the courses of concurrent chemotherapy (OR = 1.784, 95% CI 1.302–2.444, *P* < 0.001) and V_10_ of FBM_1_ (OR = 1.198, 95% CI 1.003–1.431, *P* = 0.046) were significantly associated with grade ≥ 2 anemia.

### Cutoff value of FBM_1/2_ dose-volume parameters associated with grade ≥ 2 HT

**Fig. 3:**
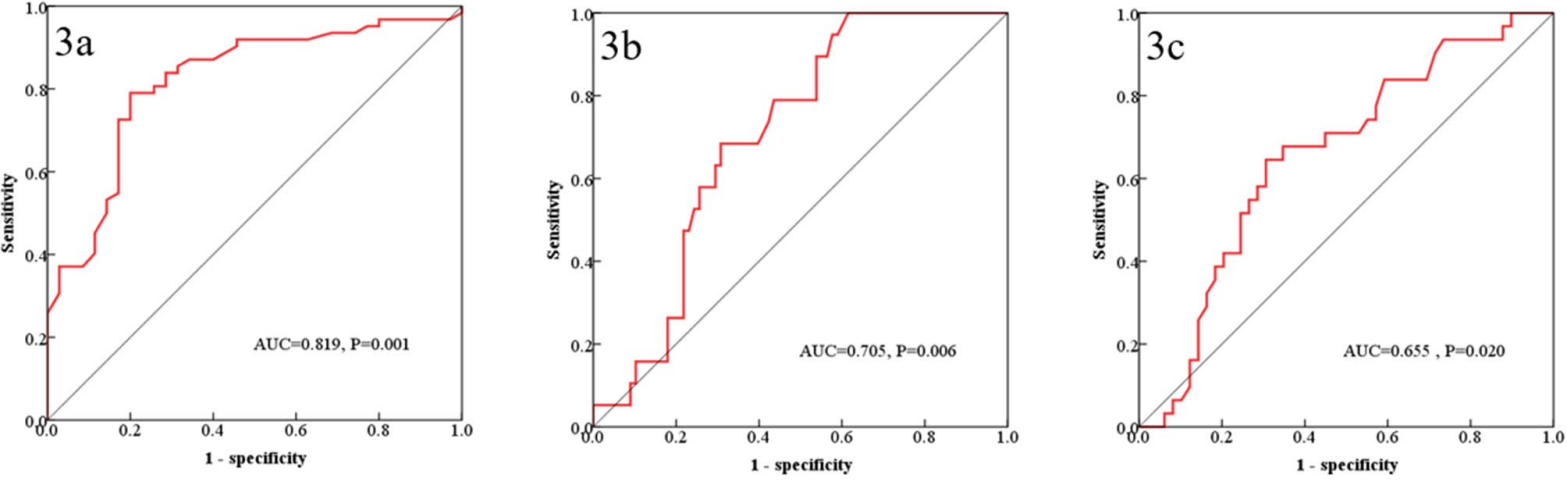
ROC curve of FBM dose-volume parameters for predicting the incidence of grade ≥ 2 HT. (**1**) ROC curve of FBM_1_ D_max_ for predicting the incidence of grade ≥ 2 leukopenia (3a). (**2**) ROC curve of FBM_2_ D_mean_ for predicting the incidence of grade ≥ 2 thrombocytopenia (3b). (**3**) ROC curve of FBM_1_ V_10_ for predicting the incidence of grade ≥ 2 anemia (3c)

To analyze the cutoff value of FBM_1/2_ dose-volume parameters, we drew the ROC curve of FBM_1_ D_max_ for predicting the incidence of grade ≥ 2 leukopenia, FBM_2_ D_mean_ for predicting the incidence of grade ≥ 2 thrombocytopenia, and FBM_1_ V_10_ for predicting the incidence of grade ≥ 2 anemia. The ROC curves are shown in Fig. 3. In the ROC curve analysis of FBM_1/2_ dose-volume parameters and HT, the largest Youden index was taken as the cutoff value. In the ROC curve analysis of the relationship between FBM_1_ D_max_ and grade ≥ 2 leukopenia, the cutoff value was 53 Gy, the sensitivity was 79%, and the specificity was 80%. In the ROC curve analysis of the relationship between FBM_2_ D_mean_ and grade ≥ 2 thrombocytopenia, the cutoff value was 33 Gy, the sensitivity was 100%, and the specificity was 38.5%. In the ROC curve analysis of the relationship between FBM_1_ V_10_ and grade ≥ 2 anemia, the cutoff value was 95%, the sensitivity was 64.5%, and the specificity was 69.4%.

The incidence of grade ≥ 2 leukopenia for patients with FBM_1_ D_max_ < 53 Gy was significantly lower than that for patients with FBM_1_ D_max_ ≥ 53 Gy (53.4% vs. 95.8%, *P* < 0.001). The incidence of grade ≥ 2 thrombocytopenia for patients with FBM_2_ D_mean_ < 33 Gy was significantly lower than that for patients with FBM_2_ D_mean_ ≥ 33 Gy (0 vs. 28.4%, *P* < 0.001). The incidence of grade ≥ 2 anemia for patients with FBM_1_ V_10_ < 95% was significantly lower than that for patients with FBM_1_ V_10_ ≥ 95% (24.4% vs. 57.1%, *P* = 0.003).

## Discussion and conclusions

It has been reported that the incidence of grade ≥ 2 HT in uterine cervical cancer during chemoradiotherapy is 23-91.37% [5, 8, 21], and the incidence of grade ≥ 3 HT is 14.7-77% [10, 14, 21, 22]. In this study, the incidence of grade ≥ 2 leukopenia, neutropenia, thrombocytopenia and anemia for patients with uterine cervical/endometrial cancer during radiotherapy was 63.9%, 45.4%, 19.6%, and 38.8%, respectively. The incidence of grade ≥ 3 leukopenia, neutropenia, thrombocytopenia and anemia was 26.8%, 14.4%, 11.3%, and 16.5%, respectively. Approximately 70% of the patients in this study received a median of four courses of concurrent chemotherapy. However, the incidence of grade ≥ 3 HT was lower than that reported in most studies. This may be attributed to the pelvic functional bone marrow being protecting well from external irradiating, and patient’s tolerance to radiotherapy and chemotherapy was improved.

To the best of our knowledge, the time of HT occurrence during radiotherapy is rarely reported in the literature. We are the first to report that the median occurrence time of grade ≥ 2 leukopenia, neutropenia, thrombocytopenia and anemia was the 29th day, 42th day, 35th day, and 31th day, respectively. The median occurrence time of grade ≥ 3 leukopenia, neutropenia, thrombocytopenia and anemia was the 38th day, 40th day, 35th day, and 41.5th day, respectively. Preclinical studies have shown that when bone marrow is exposed to a large volume of 30–40 Gy radiation, neutropenia occurs after 1 week, thrombocytopenia occurs after 2–3 weeks and hemoglobin reduction occurs after 2–3 months, while irradiation with over 50 Gy may cause irreparable damage to the microcirculation of bone marrow [1]. Therefore, it is suggested that in the 4th week of radiotherapy, patients should be alert to the occurrence of grade ≥ 2 HT, in the 5th to the 6th weeks of radiotherapy, patients should be paid more attention to the occurrence of grade ≥ 3 HT, and supportive treatment should be taken when necessary.

Radiation can lead to HT [23, 24] by damaging the pelvic bone marrow hematopoietic stem cells and bone marrow mesenchymal stem cells and causing disorders of the vascular system and bone marrow microenvironment. Pelvic bone marrow-sparing intensity-modulated radiotherapy (PBMS-IMRT) can reduce the risk for HT during radiotherapy in patients with uterine cervical cancer [4, 7, 14]. However, the delineation methods for pelvic bone marrow and the recommended dose-volume parameters are still lacking. Therefore, it is particularly important to identify active bone marrow hematopoietic regions based on imaging. The INTERTECC study [14] confirmed that PET-CT-guided PBMS-IMRT can significantly reduce the risk of grade 3 neutropenia (8.6% vs. 27.1%, *P* = 0.035), but PET-CT has a high economic cost, so the clinical application of PET-CT has been limited.

The delineation methods for pelvic bone marrow vary greatly among different studies. Most studies adopted the definition of pelvic bone marrow recommended by Mell et al. [6], who delineated the outline of the pelvic bone within the radiotherapy target volume from the top of the lumbar vertebra to the bottom of the ischial tubercle (including the bilateral femoral heads and upper segments of the femur). Mell et al. [6] collected 37 cases of uterine cervical cancer patients receiving concurrent chemotherapy and radiotherapy, and the results showed that pelvic bone marrow V_10_ was significantly related to the occurrence of grade ≥ 2 leukopenia and neutropenia, and a pelvic bone marrow V_10_ < 90% was recommended. Albuquerque et al. [5] analyzed 40 uterine cervical cancer patients who received concurrent chemoradiotherapy, and the results showed that the total pelvic bone marrow V_20_ was significantly related to the occurrence of grade ≥ 2 HT; the total pelvic bone marrow V_20_ < 80% was recommended. Rose et al. [4] analyzed 44 cases of patients with uterine cervical cancer who received adjuvant IMRT and concurrent chemotherapy after surgery, and the results showed that V_10_ and V_20_ of the total pelvic bone marrow were significantly related to the occurrence of grade ≥ 3 HT, and V_10_ < 95% and V_20_ < 76% of the total pelvic bone marrow were recommended. Wang et al. [8] analyzed 232 cases of uterine cervical cancer patients who received radiotherapy (IMRT or TOMO) and concurrent chemotherapy, and univariate analysis showed that D_max_, D_mean_, etc., of the total pelvic bone marrow were related to the occurrence of grade ≥ 2 HT, while multivariate analysis showed that V_16 − 18_, V_35_, V_36_, and V_40_ of the total pelvic bone marrow were significantly related to the occurrence of grade ≥ 2 HT. Additionally, the definition of pelvic bone marrow in the ROTG 0418 clinical trial [9] was similar to that of Mell et al. [6], who delineated all bones within the radiotherapy target volume, excluding the femoral neck and corpus femoris. This trial recruited 83 patients with gynecological tumors receiving adjuvant IMRT after surgery (40 uterine cervical cancer patients received radiotherapy and concurrent chemotherapy, and 43 endometrial cancer patients received radiotherapy alone). The results showed that the pelvic bone marrow V_40_ and D_mean_ were significantly related to the occurrence of grade ≥ 2 HT, and pelvic bone marrow V_40_ ≤ 37% and D_mean_ ≤ 34.2 Gy were recommended. Zhou et al. [10] collected 31 cases of uterine cervical cancer patients receiving concurrent chemoradiotherapy, and used FDG-PET/CT to identify active pelvic bone marrow (exceeding the systemic average SUV). The results showed that the active bone marrow V_40_ > 738 cc played a stronger role in predicting HT than values obtained through conventional pelvic bone marrow delineation.

In the multivariate regression analysis in this study, only the D_max_ of FBM_1_, V_10_ of FBM_1_ and D_mean_ of FBM_2_ were respectively significantly associated with grade ≥ 2 leukopenia, grade ≥ 2 anemia, and grade ≥ 2 thrombocytopenia. We found that patients with FBM_1_ D_max_ < 53 Gy, FBM_2_ D_mean_ < 33 Gy and FBM_1_ V_10_ < 95% had significantly lower the incidence of grade ≥ 2 HT. All these results were significantly different from those reported above, which can be mainly attributed to the outline of FBM_1/2_ and good dose constraints to the FBM_1/2_. Moreover, D_max_ of FBM_1_ had an AUC value of 0.819 for predicting grade ≥ 2 leukopenia, with the sensitivity and specificity values of approximately 80%, when the cutoff value was 53 Gy, indicating that Dmax of FBM_1_ had good prediction efficiency. As most studies had shown, Dmax of pelvic bone marrow was not related with HT. Our results also showed that there was not a statistical relationship between the Dmax of FBM_2_ and grade ≥ 2 HT. However, there was a statistical relationship between the Dmax of FBM_1_ and grade ≥ 2 leukopenia, which may be mainly due to differences in delineation of pelvic bone marrow and high heterogeneity of bone marrow hematopoiesis. The high dose irradiation of FBM_1_, which had the strongest hematopoietic function, significantly limited the hematopoietic function.

However, according to the ROC curves of FBM_2_ D_mean_ for predicting the incidence of grade ≥ 2 thrombocytopenia and FBM_1_ V_10_ for predicting the incidence of grade ≥ 2 anemia, the AUC values, the sensitivities and specificities of cutoff values were unsatisfactory. The reason may be attributed to the facts that leukopenia is the first reaction of HT, followed by thrombocytopenia and anemia during chemoradiotherapy, and the FBM2 had inferior hematopoietic ability to FBM1, which may further lessen the predictive efficiency of the dose-volume parameters.

In addition, this study found that a boost dose to the lymph nodes/parametrial tissue was an independent risk factor for grade ≥ 2 leukopenia, neutropenia and thrombocytopenia. Compared with 45 Gy/25 F of external pelvic irradiation, the prescribed dose of 50.4 Gy/28 F led to a higher risk for grade ≥ 2 leukopenia and neutropenia, which was also consistent with the finding that the high-dose volume parameters were associated with HT. Therefore, radical radiotherapy, especially when in combination with a boost dose to the lymph nodes/parametrial tissue volume, not only leads to acute HT during radiotherapy but also leads to a certain risk of late bone injury such as incomplete pelvic fracture after radiotherapy [25]. It is also well known that concurrent chemotherapy is an important factor that causes HT. Keys et al. [26] reported that the incidence of grade 3 HT for uterine cervical cancer patients in the chemoradiotherapy group was significantly higher than that in the radiotherapy alone group (21% vs. 2%). This study likewise found that the courses of concurrent chemotherapy were significantly associated with grade ≥ 2 leukopenia, neutropenia and anemia. When patients receive chemotherapy before radiotherapy, this may theoretically accelerate HT during radiotherapy. However, multifactor analyses of our study did not find the relationship between grade ≥ 2 HT and the courses of induction chemotherapy before radiotherapy, this result may be due to the limited sample size and factors such as the dose-volume parameters of FBM_1/2_, concurrent chemotherapy, and boost doses to the lymph nodes/parametrial tissue. Moreover, 30-90% of cancer patients also had anemia, and its occurrence and severity is related to patient age and disease course [27]. This study found that old age and low body weight were high-risk factors for the occurrence of grade ≥ 2 anemia during radiotherapy for uterine cervical/endometrial cancer. Therefore, concurrent chemoradiotherapy for uterine cervical /endometrial cancer patients, especially those with boost doses to the lymph nodes/parametrial tissue, is associated with a high risk of HT, and patients should be closely monitored through blood counts during radiotherapy. Elderly patients with low body weight should be given more attention to remain highly vigilant of HT in clinical practice.

Functional bone marrow protection is valuable. Pelvic bone marrow is the main hematopoietic organ of adults, accounting for approximately 40% of the whole bone marrow [28]. Bone marrow mainly divides into red bone marrow with hematopoietic function and yellow bone marrow with a large amount of adipose tissue. At present, most studies have regarded the whole bone as a dose-limiting organ and discussed the relationship between bone marrow dose-volume and HT. However, an IMRT plan that strictly limits the dose volume of the whole pelvic bone marrow may affect the coverage of the target volume and the protection of other important OARs, such as the rectum and bladder. Therefore, dose constraints to bone marrow with active hematopoietic function can better reduce the risks for HT and minimize the bad impact on the target coverage and surrounding normal organs protection. Compared with delineating the whole pelvic bones, the delineation of functional bone marrow is more conducive to shortening doctors′ manual delineation time and improving clinical efficiency.

In addition, bone marrow is also the birthplace of all kinds of immune cells, the place where B lymphocytes mature and where the humoral immune response occurs. A meta-study suggested that grade 3 lymphocytopenia induced by radiotherapy was associated with poor prognosis [29]. Therefore, pelvic radiotherapy not only damages the hematopoietic system of the body, but also may have a certain inhibitory effect on the immune system. Dose constraints for the functional bone marrow may protect immune cells as much as possible and improve the tolerance of patients to radiotherapy or chemoradiotherapy. In the current era of immunotherapy, protecting immune cells may improve the efficacy of immunotherapy and may be the research direction of pelvic radiotherapy in the future. Although this study did not discuss the relationship between FBM and lymphocytopenia, it is worth further analysis.

There were some shortcomings in this study. First, this research method was based on the functional bone marrow defined by metabolic imaging, and there were no cellular biology experiments to verify the heterogeneity of pelvic bone marrow hematopoietic function. Second, this study was a single-center, small-sample, nonrandomized controlled study, and the conclusion of this study needs to be verified by more multicenter, prospective, randomized controlled studies. Third, we did not analyze the effect of brachytherapy on myelosuppression, though this effect may be very small.

In conclusion, for patients of uterine cervical/endometrial cancer receiving radiotherapy with or without concurrent chemotherapy, grade ≥ 2 HT usually occurs in the 4th week of radiotherapy and grade ≥ 3 HT usually occurs in the 5th to 6th week of radiotherapy. The D_max_, and V_10_ of FBM_1_ and the D_mean_ of FBM_2_ were significantly associated with the occurrence of grade ≥ 2 HT. More courses of concurrent chemotherapy, a higher prescribed dose of external pelvic irradiation and a boost dose to the lymph nodes/ parametrial tissue were independent factors for an increased risk of grade ≥ 2 HT. Older age and lower weight increased the risk of grade ≥ 2 anemia. The recommended optimal dose constraints were FBM_1_ D_max_ < 53 Gy, V_10_ < 95%, and FBM_2_ D_mean_ < 33 Gy.

### Electronic supplementary material

Below is the link to the electronic supplementary material.

**Supplement Material 1**. **Supplement Table 1**. Patient treatment protocols. **Supplement Table 2**. Description statistics of FBM dose-volume parameters. **Supplement Table 3**. Single factor logistic regression analysis of grade ≥2 leukopenia. **Supplement Table 4**. Single factor logistic regression analysis of grade ≥2 neutropenia. **Supplement Table 5**. Single factor logistic regression analysis of grade ≥2 thrombocytopenia. **Supplement Table 6**. Single factor logistic regression analysis of grade ≥2 anemia.

## Data Availability

The datasets generated during the current study are available from the corresponding author on reasonable request.

## List of abbreviations

HT: Hematological toxicity
G-CSF: Granulocyte colony-stimulating factor
TPO: Thrombopoietin
IL-11: Interleukin-11
EPO: Erythropoietin
IMRT: Intensity-modulated radiation therapy
OAR: Organ at risk
FBM: Functional bone marrow
FLT-PET: 18 F-fluorothymidine positron emission tomography
SUV: Standard uptake value
GTV: Gross target volumemin
CTV: Clinical target volume
PTV: Planning target volume
IQR: Interquartile range
ROC: Receiver operating characteristic
Dmax: The maximum dose
D_mean_: The average dose
V_5_, V_10_, V_15_, V_20_, V_25_, V_30_, V_35_, V_40_, V_45_ and V_50_: the percentages of the volume receiving a dose ≥ 5 Gy, 10 Gy, 15 Gy, 20 Gy, 25 Gy, 30 Gy, 35 Gy, 40 Gy, 45 Gy, and 50 Gy, respectively
