# Supplementary material for: A prospective single-arm study on the relationship between dose-volume parameters of pelvic functional bone marrow and acute hematological toxicities during intensity-modulated radiotherapy with or without concurrent chemotherapy for uterine cervical/endometrial cancer
Source: Radiat Oncol. 2023 Nov 27;18:193. doi: 10.1186/s13014-023-02380-8 (PMC10683159; doi:10.1186/s13014-023-02380-8)
Supplement: Supplementary file 1 — Supplement Material 1. Supplement Table 1. Patient treatment protocols. Supplement Table 2. Description statistics of FBM dose-volume parameters. Supplement Table 3. Single factor logistic regression analysis of grade ≥2 leukopenia. Supplement Table 4. Single factor logistic regression analysis of grade ≥2 neutropenia. Supplement Table 5. Single factor logistic regression analysis of grade ≥2 thrombocytopenia. Supplement Table 6. Single factor logistic regression analysis of grade ≥2 anemia. [file 13014_2023_2380_MOESM1_ESM.docx]

S1 Patient treatment protocols

| Characteristic | Number（%） |
| --- | --- |
| Courses of induction chemotherapy  0  1  2  3  4 | 18（18.6）  14（14.4）  34（35.1）  22（22.7）  9（9.3） |
| Courses of concurrent chemotherapy  0  1  2  3  4  5  6  7 | 30（30.9）  5（5.2）  3（3.1）  18（18.6）  9（9.3）  24（24.7）  7（7.2）  1（1.0） |
| Interval between the last induction chemotherapy and the beginning of radiotherapy (days) | Median：51  IQR：37~70 |
| Types of radiotherapy  Postoperative adjuvant radiotherapy  Radical radiotherapy | 73（75.3）  24（24.7） |
| Boost dose to the lymph nodes/parametrial tissue  Yes  No | 18（18.6）  79（81.4） |
| Completion time of radiotherapy (days) | Median：38  IQR：36.5~42.5 |

IQR represents interquartile range.

S2 Description statistics of FBM dose-volume parameters

| Parameters | Median（IQR） | Parameters | Median（IQR） |
| --- | --- | --- | --- |
| FBM_1_ |  | FBM_2_ |  |
| V_5_ | 99.10 (97.85,99.80) | V_5_ | 99.70 (98.70,100.00) |
| V_10_ | 94.40 (92.00,96.65) | V_10_ | 97.80 (95.35,99.90) |
| V_15_ | 90.10 (83.35,93.26) | V_15_ | 94.60 (91.05,97.45) |
| V_20_ | 82.10 (75.85,88.75) | V_20_ | 88.80 (83.35,94.10) |
| V_25_ | 74.80 (68.20,79.40) | V_25_ | 78.00 (72.55,88.00) |
| V_30_ | 64.30 (57.35,69.08) | V_30_ | 68.50 (61.05,80.05) |
| V_35_ | 48.80 (42.00,54.93) | V_35_ | 54.70 (45.90,65.20) |
| V_40_ | 32.50 (25.44,37.80) | V_40_ | 37.30 (28.70,46.20) |
| V_45_ | 15.70 (8.70,21.95) | V_45_ | 22.10 (12.19,28.60) |
| V_50_ | 4.00 (0,7.20) | V_50_ | 8.90 (0,12.65) |
| D_max_ | 54.10 (48.97,55.26) | D_max_ | 54.08 (49.05,55.40) |
| D_mean_ | 32.50 (30.78,34.55) | D_mean_ | 35.00 (32.30,37.95) |

V_5_, V_10_, V_15_, V_20_, V_25_, V_30_, V_35_, V_40_, V_45_ and V_50_ represents the percentages of the FBM_1/2_ volume receiving a dose ≥ 5 Gy, 10 Gy, 15 Gy, 20 Gy, 25 Gy, 30 Gy, 35 Gy, 40 Gy, 45 Gy, and 50 Gy, respectively; Dmax represents the maximum dose to FBM_1/2_, and D_mean_ represents the average dose to FBM_1/2_; IQR represents interquartile range

S3 single factor logistic regression analysis of grade ≥2 leukopenia

| Factors | *P* | Odds ratio | 95% CI |
| --- | --- | --- | --- |
| Age | 0.127 | 0.969 | 0.925-1.010 |
| Weight | 0.508 | 0.985 | 0.942-1.030 |
| BMI | 0.192 | 0.921 | 0.814-1.042 |
| Courses of induction chemotherapy before radiotherapy | 0.002 | 1.822 | 1.243-2.671 |
| Courses of concurrent chemotherapy | ＜0.001 | 1.939 | 1.495-2.515 |
| Prescribed dose of external pelvic irradiation | ＜0.001 | 1.637 | 1.356-1.977 |
| Boost dose to the lymph nodes/ parametrial tissue（No vs. Yes） | 0.015 | 12.844 | 1.628-101.320 |
| FBM_1_ V_5_ | 0.196 | 1.174 | 0.921-1.497 |
| FBM_1_ V_10_ | 0.121 | 1.084 | 0.979-1.200 |
| FBM_1_ V_15_ | 0.497 | 0.978 | 0.915-1.044 |
| FBM_1_ V_20_ | 0.703 | 0.989 | 0.937-1.045 |
| FBM_1_ V_25_ | 0.665 | 1.011 | 0.961-1.065 |
| FBM_1_ V_30_ | 0.299 | 1.025 | 0.979-1.073 |
| FBM_1_ V_35_ | 0.039 | 1.051 | 1.002-1.102 |
| FBM_1_ V_40_ | 0.002 | 1.089 | 1.032-1.149 |
| FBM_1_ V_45_ | ＜0.001 | 1.157 | 1.077-1.242 |
| FBM_1_ V_50_ | ＜0.001 | 1.366 | 1.178-1.584 |
| FBM_1_ D_max_ | ＜0.001 | 1.467 | 1.256-1.715 |
| FBM_1_ D_mean_ | 0.058 | 1.156 | 0.995-1.344 |
| FBM_2_ V_5_ | 0.004 | 2.027 | 1.259-3.264 |
| FBM_2_ V_10_ | 0.009 | 1.265 | 1.060-1.509 |
| FBM_2_ V_15_ | 0.293 | 1.040 | 0.967-1.119 |
| FBM_2_ V_20_ | 0.547 | 1.017 | 0.963-1.074 |
| FBM_2_ V_25_ | 0.192 | 1.028 | 0.986-1.073 |
| FBM_2_ V_30_ | 0.040 | 1.036 | 1.002-1.075 |
| FBM_2_ V_35_ | 0.014 | 1.041 | 1.008-1.074 |
| FBM_2_ V_40_ | 0.014 | 1.045 | 1.009-1.081 |
| FBM_2_ V_45_ | 0.007 | 1.060 | 1.016-1.106 |
| FBM_2_ V_50_ | 0.001 | 1.131 | 1.052-1.215 |
| FBM_2_ D_max_ | ＜0.001 | 1.398 | 1.211-1.613 |
| FBM_2_ D_mean_ | 0.008 | 1.189 | 1.046-1.350 |

S4 single factor logistic regression analysis of grade ≥2 neutropenia

| Factors | *P* | Odds ratio | 95% CI |
| --- | --- | --- | --- |
| Age | 0.474 | 0.985 | 0.945-1.027 |
| Weight | 0.704 | 0.992 | 0.949-1.036 |
| BMI | 0.289 | 0.936 | 0.828-1.058 |
| Courses of induction chemotherapy before radiotherapy | 0.026 | 1.490 | 1.049-2.116 |
| Courses of concurrent chemotherapy | ＜0.001 | 1.881 | 1.460-2.424 |
| Prescribed dose of external pelvic irradiation | ＜0.001 | 1.836 | 1.414-2.238 |
| Boost dose to the lymph nodes/ parametrial tissue（No vs. Yes） | 0.001 | 14.571 | 3.122-68.008 |
| FBM_1_ V_5_ | 0.172 | 1.203 | 0.923-1.568 |
| FBM_1_ V_10_ | 0.299 | 1.055 | 0.953-1.169 |
| FBM_1_ V_15_ | 0.190 | 0.959 | 0.900-1.021 |
| FBM_1_ V_20_ | 0.301 | 0.972 | 0.922-1.025 |
| FBM_1_ V_25_ | 0.976 | 0.999 | 0.951-1.050 |
| FBM_1_ V_30_ | 0.463 | 1.017 | 0.973-1.063 |
| FBM_1_ V_35_ | 0.093 | 1.038 | 0.994-1.084 |
| FBM_1_ V_40_ | 0.018 | 1.059 | 1.010-1.111 |
| FBM_1_ V_45_ | 0.001 | 1.105 | 1.043-1.172 |
| FBM_1_ V_50_ | ＜0.001 | 1.237 | 1.106-1.384 |
| FBM_1_ D_max_ | ＜0.001 | 1.480 | 1.255-1.745 |
| FBM_1_ D_mean_ | 0.232 | 1.087 | 0.948-1.246 |
| FBM_2_ V_5_ | 0.001 | 2.615 | 1.451-4.714 |
| FBM_2_ V_10_ | 0.005 | 1.306 | 1.082-1.576 |
| FBM_2_ V_15_ | 0.112 | 1.065 | 0.985-1.151 |
| FBM_2_ V_20_ | 0.179 | 1.038 | 0.983-1.097 |
| FBM_2_ V_25_ | 0.126 | 1.033 | 0.991-1.076 |
| FBM_2_ V_30_ | 0.046 | 1.034 | 1.001-1.068 |
| FBM_2_ V_35_ | 0.024 | 1.035 | 1.005-1.066 |
| FBM_2_ V_40_ | 0.015 | 1.039 | 1.008-1.072 |
| FBM_2_ V_45_ | 0.004 | 1.059 | 1.018-1.101 |
| FBM_2_ V_50_ | ＜0.001 | 1.141 | 1.064-1.224 |
| FBM_2_ D_max_ | ＜0.001 | 1.411 | 1.217-1.635 |
| FBM_2_ D_mean_ | 0.007 | 1.176 | 1.045-1.324 |

S5 single factor logistic regression analysis of grade ≥2 [thrombocytopenia](javascript:;)

| Factors | *P* | Odds ratio | 95% CI |
| --- | --- | --- | --- |
| Age | 0.609 | 1.014 | 0.963-1.067 |
| Weight | 0.075 | 0.943 | 0.884-1.006 |
| BMI | 0.037 | 0.822 | 0.683-0.988 |
| Courses of induction chemotherapy before radiotherapy | 0.299 | 1.252 | 0.819-1.915 |
| Courses of concurrent chemotherapy | 0.002 | 1.608 | 1.188-2.177 |
| Prescribed dose of external pelvic irradiation | 0.013 | 1.634 | 1.109-2.406 |
| Boost dose to the lymph nodes/ parametrial tissue（No vs. Yes） | ＜0.001 | 9.722 | 3.047-31.019 |
| FBM_1_ V_5_ | 0.617 | 1.086 | 0.787-1.499 |
| FBM_1_ V_10_ | 0.489 | 1.049 | 0.917-1.199 |
| FBM_1_ V_15_ | 0.743 | 0.987 | 0.914-1.066 |
| FBM_1_ V_20_ | 0.947 | 0.998 | 0.934-1.066 |
| FBM_1_ V_25_ | 0.609 | 1.016 | 0.956-1.080 |
| FBM_1_ V_30_ | 0.299 | 1.030 | 0.974-1.089 |
| FBM_1_ V_35_ | 0.171 | 1.037 | 0.985-1.092 |
| FBM_1_ V_40_ | 0.043 | 1.060 | 1.002-1.121 |
| FBM_1_ V_45_ | 0.015 | 1.084 | 1.016-1.157 |
| FBM_1_ V_50_ | 0.004 | 1.197 | 1.060-1.350 |
| FBM_1_ D_max_ | 0.001 | 1.313 | 1.126-1.531 |
| FBM_1_ D_mean_ | 0.197 | 1.117 | 0.944-1.321 |
| FBM_2_ V_5_ | 0.022 | 3.560 | 1.206-10.511 |
| FBM_2_ V_10_ | 0.058 | 1.273 | 0.992-1.634 |
| FBM_2_ V_15_ | 0.071 | 1.117 | 0.990-1.261 |
| FBM_2_ V_20_ | 0.052 | 1.082 | 0.999-1.171 |
| FBM_2_ V_25_ | 0.026 | 1.064 | 1.007-1.124 |
| FBM_2_ V_30_ | 0.019 | 1.052 | 1.008-1.098 |
| FBM_2_ V_35_ | 0.038 | 1.038 | 1.002-1.076 |
| FBM_2_ V_40_ | 0.080 | 1.031 | 0.996-1.067 |
| FBM_2_ V_45_ | 0.053 | 1.040 | 0.999-1.083 |
| FBM_2_ V_50_ | 0.011 | 1.114 | 1.025-1.212 |
| FBM_2_ D_max_ | 0.002 | 1.246 | 1.085-1.430 |

S6 single factor logistic regression analysis of grade ≥2 anemia

| Factors | *P* | Odds ratio | 95% CI |
| --- | --- | --- | --- |
| Age | 0.072 | 1.047 | 0.996-1.101 |
| Weight | 0.074 | 0.949 | 0.895-1.005 |
| BMI | 0.068 | 0.862 | 0.725-1.011 |
| Courses of induction chemotherapy before radiotherapy | 0.038 | 1.526 | 1.024-2.273 |
| Courses of concurrent chemotherapy | 0.001 | 1.556 | 1.205-2.009 |
| Prescribed dose of external pelvic irradiation | 0.002 | 1.439 | 1.140-1.817 |
| Boost dose to the lymph nodes/ parametrial tissue（No vs. Yes） | 0.002 | 8.433 | 2.121-35.533 |
| FBM_1_ V_5_ | 0.055 | 1.418 | 0.992-2.026 |
| FBM_1_ V_10_ | 0.044 | 1.148 | 1.004-1.314 |
| FBM_1_ V_15_ | 0.729 | 1.012 | 0.946-1.082 |
| FBM_1_ V_20_ | 0.937 | 1.002 | 0.946-1.062 |
| FBM_1_ V_25_ | 0.961 | 1.001 | 0.938-1.057 |
| FBM_1_ V_30_ | 0.314 | 1.026 | 0.976-1.077 |
| FBM_1_ V_35_ | 0.127 | 1.037 | 0.990-1.087 |
| FBM_1_ V_40_ | 0.033 | 1.057 | 1.005-1.112 |
| FBM_1_ V_45_ | 0.031 | 1.065 | 1.006-1.127 |
| FBM_1_ V_50_ | 0.051 | 1.114 | 1.000-1.241 |
| FBM_1_ D_max_ | 0.001 | 1.268 | 1.102-1.459 |
| FBM_1_ D_mean_ | 0.102 | 1.133 | 0.975-1.316 |
| FBM_2_ V_5_ | 0.011 | 2.371 | 1.222-4.598 |
| FBM_2_ V_10_ | 0.008 | 1.357 | 1.082-1.702 |
| FBM_2_ V_15_ | 0.078 | 1.086 | 0.991-1.192 |
| FBM_2_ V_20_ | 0.225 | 1.039 | 0.997-1.106 |
| FBM_2_ V_25_ | 0.479 | 1.016 | 0.972-1.062 |
| FBM_2_ V_30_ | 0.321 | 1.018 | 0.983-1.053 |
| FBM_2_ V_35_ | 0.316 | 1.016 | 0.985-1.048 |
| FBM_2_ V_40_ | 0.318 | 1.016 | 0.985-1.048 |
| FBM_2_ V_45_ | 0.160 | 1.027 | 0.989-1.066 |
| FBM_2_ V_50_ | 0.028 | 1.084 | 1.009-1.165 |
| FBM_2_ D_max_ | 0.001 | 1.238 | 1.085-1.413 |
| FBM_2_ D_mean_ | 0.143 | 1.094 | 0.970-1.232 |
